# Frequency of *KCNQ1* variants causing loss of methylation of Imprinting Centre 2 in Beckwith-Wiedemann syndrome

**DOI:** 10.1186/s13148-020-00856-y

**Published:** 2020-05-11

**Authors:** Carla Eßinger, Stephanie Karch, Ute Moog, György Fekete, Anna Lengyel, Eva Pinti, Thomas Eggermann, Matthias Begemann

**Affiliations:** 1 Institute of Human Genetics, Medical Faculty, RWTH Aachen University, Pauwelsstr. 30, 52074 Aachen, Germany; 2 University Children’s Hospital, Heidelberg University, Heidelberg, Germany; 3 Institute of Human Genetics, Heidelberg University, Heidelberg, Germany; 4 II. Department of Paediatrics, Semmelweis University, Budapest, Hungary

**Keywords:** Beckwith-Wiedemann syndrome, *KCNQ1* variants, Imprinting Centre 2, Loss of methylation, Long-QT syndrome

## Abstract

**Background:**

Beckwith-Wiedemann syndrome (BWS) is an imprinting disorder caused by disturbances of the chromosomal region 11p15.5. The most frequent molecular finding in BWS is loss of methylation (LOM) of the Imprinting Centre 2 (IC2) region on the maternal allele, which is localised in intron 10 of the *KCNQ1* gene. In rare cases, LOM of IC2 has been reported in families with *KCNQ1* germline variants which additionally cause long-QT syndrome (LQTS). Thus, a functional link between disrupted *KCNQ1* transcripts and altered IC2 methylation has been suggested, resulting in the co-occurrence of LQTS and BWS in case of maternal inheritance. Whereas these cases were identified by chance or in patients with abnormal electrocardiograms, a systematic screen for *KCNQ1* variants in IC2 LOM carriers has not yet been performed.

**Results:**

We analysed 52 BWS patients with IC2 LOM to determine the frequency of germline variants in *KCNQ1* by MLPA and an amplicon-based next generation sequencing approach. We identified one patient with a splice site variant causing premature transcription termination of *KCNQ1*.

**Conclusions:**

Our study strengthens the hypothesis that proper *KCNQ1* transcription is required for the establishment of IC2 methylation, but that *KCNQ1* variants cause IC2 LOM only in a small number of BWS patients.

## Background

Beckwith-Wiedemann syndrome (BWS, OMIM #130650) is a congenital imprinting disorder which was first described by Beckwith and Wiedemann in 1963 [1, 2]. Clinically, it presents with features including macroglossia, exomphalos, lateralised overgrowth, an increased risk for Wilms tumour, prolonged hyperinsulinism after birth and several other symptoms (for review: [3]). In the past, the phenotypic heterogeneity has hindered the clinical diagnosis; therefore, in 2018, Brioude et al. have introduced a consensus scoring system that allows the classification of patients with BWS according to the symptoms and the molecular alteration [3].

BWS is associated with disturbances of the chromosomal region 11p15.5 which harbours two imprinting control regions [4]. The Imprinting Centre 1 (IC1; H19/IGF2:IG-DMR) is paternally methylated and regulates the monoallelic expression of the *insulin-like growth factor 2* (*IGF2*) and *H19* genes. The Imprinting Centre 2 (IC2; KCNQ1OT1:TSS-DMR) controls the expression of the genes *KCNQ1*, *KCNQ1OT1* and *CDKN1C*. In contrast to the IC1, the IC2 is maternally methylated leading to an expression of *KCNQ1* and *CDKN1C* and a repression of *KCNQ1OT1* on the maternal allele, while it is unmethylated on the paternal allele resulting in silencing of *KCNQ1* and *CDKN1C* and expression of *KCNQ1OT1* (for review: [5]).

The most frequent molecular cause of BWS is loss of methylation (LOM) of the IC2 (50–60% of patients) causing a biallelic expression of *KCNQ1OT1* and repression of maternally expressed genes [4]. Further alterations comprise a paternal uniparental disomy (UPD) of chromosome 11p15.5 (20–25%) and a gain of methylation (GOM) at the IC1 (~ 4%) (for review: [3]). Point mutations of *CDKN1C* are rare in sporadic BWS (1.3–5%) but more common in familial cases of BWS (20–40%) [6]. Other molecular cases such as copy number variants (CNVs) of the IC1 and/or IC2 only occur in single cases (< 1%) [7]. An epigenotype-phenotype correlation could be delineated for some symptoms, such as omphalocele, hemihypertrophy and the risk to develop neoplasias [8].

BWS mostly occurs sporadically, but in addition to inherited variants in *CDKN1C* [9], familial occurrence has also been described for chromosomal rearrangements and CNVs in IC1 and IC2 [6], among them deletions within the *KCNQ1* gene [10–13]. Some of them include the IC2 domain itself and are therefore associated with a LOM of the IC2. However, there are also cases where a loss of transcription of *KCNQ1*, either by CNVs or single nucleotide variants (SNVs), causes a LOM of the IC2 [14–16]. Remarkably, these genetic alterations affect only *KCNQ1* and its regulatory elements, but not the IC2 itself. In mice, a maternally inherited poly(A) cassette, inserted into *Kcnq1*, terminates the transcription of *Kcnq1* upstream of IC2 and results in LOM of the IC2, biallelic expression of *Kcnq1ot1* and silencing of *Cdkn1c* and *Kcnq1* [17].

Loss-of-function mutations in *KCNQ1* are the most frequent causes for congenital long-QT syndrome (LQTS) [18], a hereditary cardiac disease with a prolonged QT interval resulting in a significant risk of arrhythmias and sudden death (for review: [19]). The penetrance of LQTS is variable as only about 60% of carriers of a pathogenic *KCNQ1* variant have a prolonged QT interval. Vice versa, 10% of the genetically affected patients who do not have a prolonged QT interval can be affected by a cardiac event until the age of 40 years [20]. It is conceivable that apparently sporadic BWS cases with IC2 LOM are caused by familial pathogenic *KCNQ1* variants but remained undetected so far due to the reduced penetrance of LQTS variants. Up to now, six LQTS families have been described in which altered *KCNQ1* transcripts lead to BWS in combination with LQTS when inherited from the mother or to isolated LQTS when transmitted from the father [14, 21–23]. The different consequences depend on the parental origin of the allele carrying the altered *KCNQ1* variant and can therefore be explained by the biallelic expression of *KCNQ1* in the heart but monoallelic expression in other tissues [21]. However, these were mainly case reports and a systematic screen for *KCNQ1* variants in BWS patients associated with IC2 LOM is missing.

In our study, we therefore aimed to determine the frequency of pathogenic germline variants in *KCNQ1* among BWS patients with IC2 LOM.

## Results

Copy number analysis of the coding sequence of *KCNQ1* by multiplex ligation-dependent probe amplification (MLPA) did not reveal any deletion or duplication in the cohort of 52 BWS patients with IC2 LOM.

**Fig. 1:**
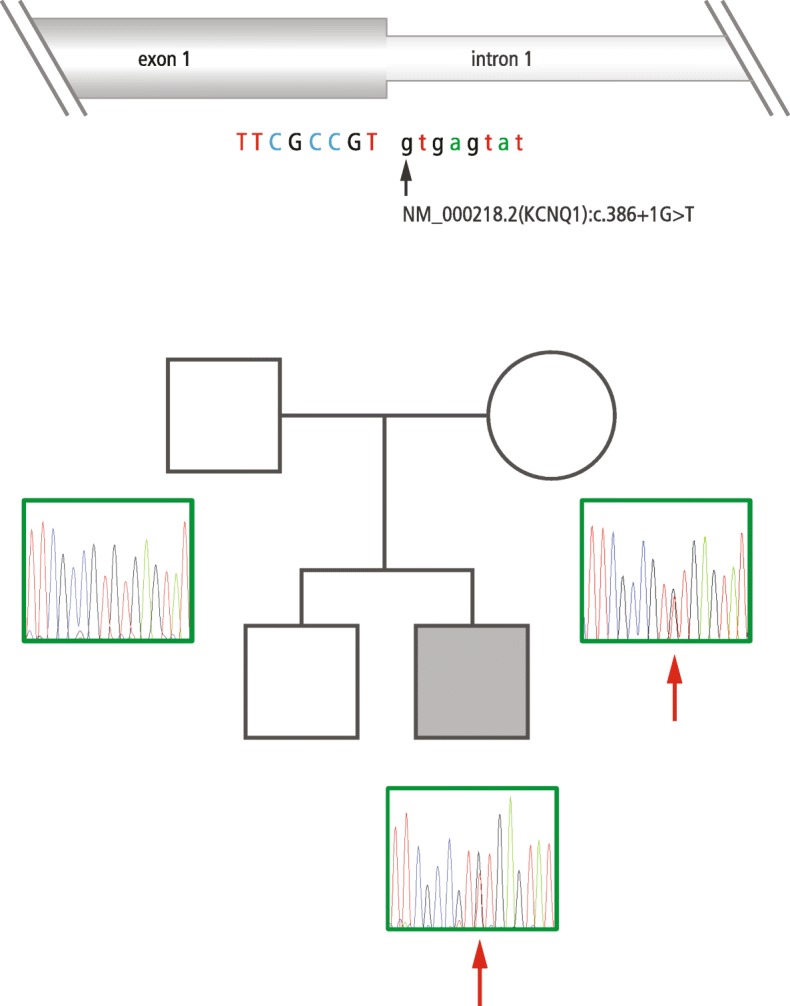
Sequencing results of the family with the donor splice site variant in intron 1 of the *KCNQ1* gene. The patient and the patient’s mother carry the splice site variant in intron 1 of *KCNQ1* (NM_000218.2(KCNQ1):c.386+1G>T). The patient is diagnosed with BWS due to IC2 LOM. The mother and the brother of the patient are healthy

By an amplicon-based next generation sequencing (NGS) approach, heterozygosity for a splice variant in *KCNQ1* was detected in one patient. The variant was located in the donor splice site of intron 1 of *KCNQ1* (NM_000218.2:c.386+1G>T; Chr11:2466715G>T, HG19). The change was also detected in the mother but not in the father. The variant affects the canonical donor splice site of intron 1 (MaxEnt, NNSplice, SSF, − 100%) (Fig. 1) and is therefore predicted to impair the transcription of *KCNQ1*.

### Patient with IC2 LOM and KCNQ1 variant

The boy was born at 26 weeks of gestation by caesarean section due to premature placental separation and preeclampsia with a birthweight of 950 g (50th percentile), a length of 33 cm (30th percentile) and a head circumference of 23 cm (15th percentile). He showed marked macroglossia, cleft palate, umbilical and inguinal hernias, hypospadias, ear lobe creases and a nevus flammeus in his face. He suffered from chronic respiratory insufficiency due to bronchopulmonary dysplasia as a consequence of his prematurity. He underwent multiple surgeries during the first months of life, including reduction of macroglossia. He showed a severe feeding disorder and required tube feeding for several years. His psychomotor development was significantly delayed putatively due to his premature birth. At the age of 3 years, he was able to walk without support, and he started speaking a few words at the age of 7 years. On the last examination at the age of eight and a half years, he was attending a school for special needs. His vision was restricted because of retinopathy of prematurity, and he needed regular controls for a nephrocalcinosis °I–II.

Clinical diagnosis of BWS was confirmed by molecular detection of IC2 LOM at the age of 2 months. Molecular karyotyping did not reveal any CNVs (resolution of 50 kb). He had one healthy brother. His mother was healthy as well and did not show any BWS features. MLPA of the mother showed a normal methylation pattern.

An electrocardiography (ECG) of the patient was performed after the results of the molecular analysis of *KCNQ1* and revealed a borderline prolonged QTc interval of 440–450 ms. The long-term ECG showed normal findings but sporadic extrasystoles. The mother’s ECG revealed no pathologic findings.

## Discussion

**Fig. 2:**
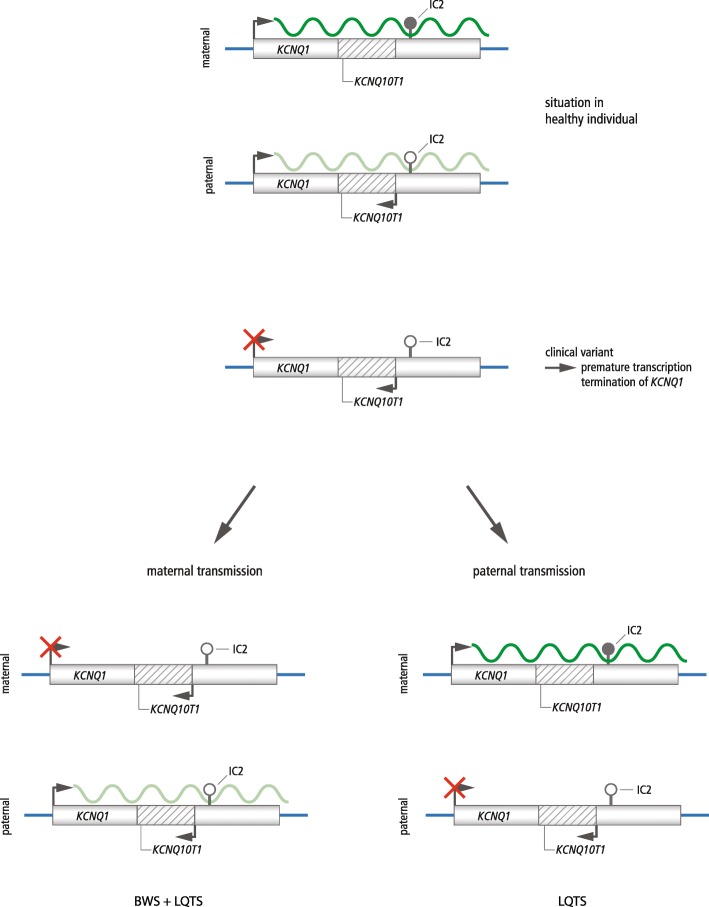
Hypothesised model of the effect of premature transcription termination of *KCNQ1* on the methylation status of the IC2 (figure modified from Valente et al. [21]). In healthy individuals, IC2 is methylated on the maternal allele (indicated by a filled lollipop) leading to an expression of *KCNQ1* and a repression of *KCNQ1OT1.* On the unmethylated paternal allele (indicated by an open lollipop), *KCNQ1* is repressed but *KCNQ1OT1* is expressed. Active promoters and transcription are indicated by bent black arrows, and correct transcription of *KCNQ1* is depicted by a curved dark green line. The curved light green line indicates the paternal transcription of *KCNQ1* in the heart. Due to a variant in *KCNQ1*, the transcription of *KCNQ1* is disturbed (indicated by a red cross). After maternal transmission, the variant leads to BWS due to the LOM of IC2 and, due to haploinsufficiency of *KCNQ1*, to LQTS. After paternal transmission, the variant may lead to isolated LQTS due to the haploinsufficiency of *KCNQ1*

By analysing 52 BWS patients with IC2 LOM for *KCNQ1* germline variants, we identified one case with a maternally inherited splice site variant (c.386+1G>T) affecting the splice donor site of intron 1 of *KCNQ1*. A variant at the same position with another base pair substitution (c.386 +1G>C) has been described by Valente et al. [21] in a BWS patient with IC2 LOM (patient 2). This patient showed typical BWS features and was diagnosed with LQTS on the basis of an ECG performed after an episode of chest pain. The patient’s mother did not show any signs of BWS but was diagnosed as asymptomatic LQTS with a prolonged QT interval [21]. The authors suggested that the splice site variant leads to a premature transcription termination and a disturbed elongation of the *KCNQ1* transcript. The resulting monoallelic expression of *KCNQ1* could explain the LQTS phenotype as *KCNQ1* is expressed biallelically in the heart [21]. In tissues with monoallelic expression of the maternal allele, the altered transcript should cause a LOM of the IC2 (Fig. 2).

Five further cases corroborate the hypothesis that interrupted transcription of *KCNQ1* upstream of IC2 results in LOM. A deletion in *KCNQ1* has been reported that does not include the IC2 but causes LOM, suggesting that transcription of the entire *KCNQ1* gene is required for the establishment of the imprinting mark at the IC2 locus [14]. Demars et al. described a 50-kb duplication in *KCNQ1* upstream of IC2 which leads to an IC2 LOM and BWS symptoms when inherited from the mother but to a normal phenotype in case of paternal inheritance [15]. In a third family, a translocation with a breakpoint in intron 9 of *KCNQ1* results in a LOM of the IC2 and BWS when maternally inherited [16]. Valente et al. described two additional patients: one with a deletion in exon 1 of *KCNQ1* and one with a 160-kb *KCNQ1* duplication. Both patients were affected by LQTS and BWS due to an IC2 LOM [21]. In our case, LQTS could not be detected in the patient’s mother but this finding can be explained by the variable penetrance of LQTS [20]. The borderline prolonged QTc interval associated with the probably pathogenic variant found in the patient makes LQTS in the patient very likely.

In fact, functional analyses have not been conducted but the effect of the splice site variant can be delineated from findings published recently by Valente et al. [21].

Up to now, only case reports and CNV studies have been published, but systematic studies to determine the frequency of *KCNQ1* variants in BWS patients with IC2 LOM have not yet been performed. Demars et al. screened 78 BWS patients with IC2 LOM for CNVs and discovered one patient with a duplication including exon 2 and parts of intron 1 and 2 of *KCNQ1*. However, they did not perform ECG examinations [15]. Similarly, Baskin et al. discovered two out of 103 BWS patients with CNVs within the IC2 region but ECG data were not provided [13]. Valente et al. restricted their mutation analysis to three BWS patients with ECG abnormalities and therefore might have missed *KCNQ1* mutation carriers without a QT prolongation [21]. In addition to these studies, single case reports on BWS families with *KCNQ1* germline variants and multiple miscarriages [14, 16] or episodes of cardiac arrhythmia [22, 23] have been published. Other case reports describe further deletions in *KCNQ1* [10–12]. Thus, this is the first systematic screening of a cohort of BWS patients with IC2 LOM for pathogenic variants in the *KCNQ1* gene. However, our screening report has some technical limitations. The used MLPA kit does not cover exon 5 of *KCNQ1* (see supplementary table); furthermore, deletions outside of the MLPA probe’s hybridisation sites might escape detection. The amplicon-based NGS approach only identifies exonic variants while variants in non-coding regions may not be covered by the primers (see supplementary table).

Altogether, only a small number of patients with both functional relevant *KCNQ1* variants and IC2 LOM have been identified; therefore, the frequency of BWS patients at risk for *KCNQ1*-related LQTS appears to be low. However, it can be assumed that the frequency of *KCNQ1* germline variants in BWS might be higher due to incomplete penetrance of *KCNQ1* variants. In fact, about 40% of carriers of pathogenic *KCNQ1* variants do not exhibit a prolonged QT interval [20]. However, it is important to identify patients with a *KCNQ1* variant that may lead to LQTS, as these individuals are at risk of developing cardiac events even when asymptomatic. This also applies to relatives of these patients in case they are carriers of the variant. Furthermore, cardiac events can also occur in genetically affected persons without QT prolongation [20].

The identification of *KCNQ1* variants leading to an altered transcription of *KCNQ1* is relevant not only for the prediction of LQTS but also for the recurrence risk of BWS. The majority of BWS cases occur sporadically, but in case of IC2 LOM caused by a germline variant in *KCNQ1*, the probability of transmission and the risk of offspring suffering from BWS and LQTS are increased [13]. The risk of transmission via the maternal germline and therefore to suffer from BWS is 50%, whereas it is generally 50% for LQTS regardless of the parental origin of the allele.

## Conclusions

The correlation between BWS and LQTS has an impact on the clinical recommendations and surveillance of the patients with both diseases. Early diagnosis of LQTS is important to prevent life-threatening cardiac arrhythmias. The first line therapy of LQTS is a treatment with β-blockers (e.g. propranolol). In case of therapy refractoriness, left cardiac sympathetic denervation or an implantable cardioverter defibrillator (ICD) is necessary. Also, the patients should avoid competitive sports (e.g. swimming) and the intake of QT interval prolonging drugs (for review: [19]). The precise molecular diagnosis of BWS is important for the clinical management, the screening protocol and the estimation of the recurrence risk. An international consensus group has already recommended to perform an ECG in patients with CNVs involving the IC2 region [3]. However, screening for *KCNQ1* variants is not carried out routinely and SNVs may escape routine diagnostic detection. As screening for *KCNQ1* variants in all IC2 LOM patients would be expensive, an ECG in this group of patients is a suitable examination tool and should be performed in every patient with IC2 LOM.

## Methods

### Patients

We investigated 52 BWS patients carrying an IC2 LOM. The molecular diagnosis had been confirmed by methylation-specific multiplex ligation-dependent probe amplification (MS-MLPA; assay ME030, MRC-Holland, Amsterdam, The Netherlands) according to the manufacturer’s instructions. Multilocus imprinting disturbances (MLID) were excluded by screening with a MS-MLPA-based multilocus assay (assay ME034, MRC-Holland). Patients with MLID were excluded as they are probably caused by other (genomic) alterations than by *KCNQ1* variants. The study was approved by the ethical committee of the Medical Faculty of the RWTH Aachen University (EK303-18).

### CNV analysis of the KCNQ1 gene

The patient’s DNA samples were analysed for deletions or duplications of the *KCNQ1* gene by MLPA (assay P114-B3-0517, MRC-Holland) following the manufacturer’s instructions.

### Variant detection by amplicon-based next generation sequencing

**Fig. 3:**
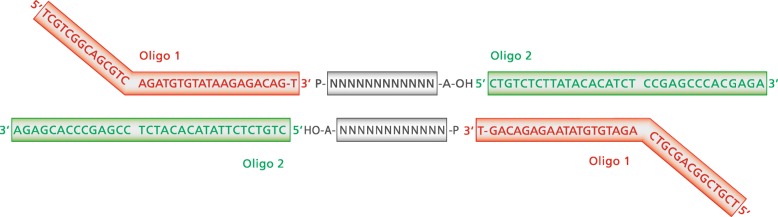
Design of the amplicon-based NGS analysis of the coding region of *KCNQ1* (based on the sequences of transposase oligos published in Bogdanoff et al. [24]). After the phosphorylation and annealing of the oligo adapters, the adapters bind to the PCR products which are prepared with 3′-A overhangs

All patients were screened for SNVs in *KCNQ1* by an amplicon-based next generation sequencing approach. First, the exons of the whole coding region of *KCNQ1* were amplified by polymerase chain reaction (PCR); primers and PCR conditions are listed in supplementary tables. The 17 PCR assays were multiplexed; finally, three PCRs were performed per sample. For the amplification of exon 1, a nested PCR assay was used (see supplementary tables). PCR products of the same individual were pooled, and 3′-A overhangs were added to the PCR products by using the NEBNext Ultra II End Prep Enzyme Mix (New England Biolabs, Ipswich, USA) to allow the binding of the specifically designed oligo adapters (see supplementary tables). The adapters are based on the sequences of the transposase oligos published in Bogdanoff et al. [24] (Fig. 3). These oligos were annealed and phosphorylated using the T4 Polynucleotide Kinase (New England Biolabs) (see supplementary tables) and subsequently ligated to the PCR products, using the Blunt/TA Ligase Master Mix (New England Biolabs) (see supplementary tables). The ligated oligo adapters serve as connectors between the PCR product and the Nextera index primers (Illumina, San Diego, USA). To add the sequencing adapters and to index the samples, a second PCR with the pooled PCR products and the Illumina Nextera Index Adapter N70X and E50X was performed using the KAPA Hifi Ready Mix (Roche Sequencing and Life Science, Kapa Biosystems, Wilmington, USA) (see supplementary tables). The concentration of the final library was determined by quantitative PCR and sequenced on a MiSeq Nano Flowcell (Illumina, San Diego, USA) with 2 × 150 cycles. FastQ files were generated by the built-in standard MiSeq pipeline. Data analysis and variant calling were performed with SeqNext (JSI medical systems, Ettenheim, Germany). Synonymous variants and variants with a frequency > 1% in public databases were discarded. Variant confirmation and segregational analyses were conducted by Sanger sequencing.

## Supplementary information

**Additional file 1: Supplementary tables.** Primer sequences and PCR conditions for the amplicon-based NGS analysis of *KCNQ1.*

## Data Availability

The datasets generated and/or analysed during the current study are not publicly available due to privacy restrictions but are available from the corresponding author on reasonable request.

## Abbreviations

BWS: Beckwith-Wiedemann syndrome
CNV: Copy number variant
ECG: Electrocardiogram
GOM: Gain of methylation
IC1: Imprinting Centre 1 in 11p15.5
IC2: Imprinting Centre 2 in 11p15.5
LOM: Loss of methylation
LQTS: Long-QT syndrome
MLID: Multilocus imprinting disturbances
MLPA: Multiplex ligation-dependent probe amplification
MS-MLPA: Methylation-specific multiplex ligation-dependent probe amplification
NGS: Next generation sequencing
SNV: Single nucleotide variant
