# Supplementary material for: Frequency of KCNQ1 variants causing loss of methylation of Imprinting Centre 2 in Beckwith-Wiedemann syndrome
Source: Clin Epigenetics. 2020 May 11;12:63. doi: 10.1186/s13148-020-00856-y (PMC7216698; doi:10.1186/s13148-020-00856-y)
Supplement: Supplementary file 1 — Additional file 1: Supplementary tables. Primer sequences and PCR conditions for the amplicon-based NGS analysis of KCNQ1. [file 13148_2020_856_MOESM1_ESM.docx]

**Supplementary table 1a: Physical positions of MLPA probes and NGS primers (GRCh37/hg19)**

| Exon | Physical position | MLPA probes | Primers for NGS |
| --- | --- | --- | --- |
| Exon 1 | Chr11:2466329-2466714 | Chr11:2466643-2466700 | Exon 1 full: Chr11:2465996-2467013 |
|  |  |  | Exon 1.1:  Chr11:2466274-2466484 |
|  |  |  | Exon 1.2:  Chr11:2466426-2466616 |
|  |  |  | Exon1.3:  Chr11:2466549-2466756 |
| altExon 1 | Chr11:2482684-2482944 | Chr11:2482860-2482917 | Chr11:2482806-2483037 |
| Exon 2 | Chr11:2549158-2549248 | Chr11:2549164-2549220 | Chr11:2549126-2549306 |
| Exon 3 | Chr11:2591858-2591984 | Chr11:2591907-2591959 | Chr11:2591797-2592013 |
| Exon 4 | Chr11:2592555-2592633 | Chr11:2592556-2592608 | Chr11:2592480-2592693 |
| Exon 5 | Chr11:2593243-2593339 |  | Chr11:2593212-2593386 |
| Exon 6 | Chr11:2594076-2594216 | Chr11:2594087-2594155 | Chr11:2594039-2594245 |
| Exon 7 | Chr11:2604665-2604775 | Chr11:2604702-2604756 | Chr11:2604636-2604819 |
| Exon 8 | Chr11:2606442-2606537 | Chr11:2606444-2606507 | Chr11:2606397-2606573 |
| Exon 9 | Chr11:2608800-2608922 | Chr11:2608805-2608861 | Chr11:2608758-2608967 |
| Exon 10 | Chr11:2609943-2610084 | Chr11:2609962-2610030 | Chr11:2609908-2610139 |
| Exon 11 | Chr11:2683191-2683311 | Chr11:2683179-2683248 | Chr11:2683155-2683354 |
| Exon 12 | Chr11:2790074-2790149 | Chr11:2790057-2790118 | Chr11:2790022-2790180 |
| Exon 13 | Chr11:2797190-2797284 | Chr11:2797237-2797288 | Chr11:2797116-2797354 |
| Exon 14 | Chr11:2798216-2798262 | Chr11:2798205-2798271 | Chr11:2798176-2798292 |
| Exon 15 | Chr11:2799206-2799267 | Chr11:2799204-2799265 | Chr11:2799170-2799316 |
| Exon 16 | Chr11:2868997-2869233 | Chr11:2869130-2869184  Chr11:2869986-2870048 | Exon 16.1:  Chr11:2868955-2869173 |
|  |  |  | Exon 16.2:  Chr11:2869052-2869270 |

**Supplementary table 1b: Primers**

| Exon | F-Primer | R-Primer | Product size |
| --- | --- | --- | --- |
| Exon 1.1 | GCTGCAGCTCCCGGTGCC | CGCGTAGAGCGCGCCGCC | 211 bp |
| Exon 1.2 | AGTGCCCCTTCTCGCTG | CGTGCTGTAGATGGAGACGC | 191 bp |
| Exon 1.3 | CAGTTGCCTCCGACCTTG | GGAAGCACCTTCGTGCC | 208 bp |
| Exon 1 full | GACAGGCCAAGCCAGGG | TCACACCTGGACTACCCTCC | 1018 bp |
| altExon 1 | ACGGCTGCTTTTGTTTACGT | ATCTACCCGCCACCCATAAC | 232 bp |
| Exon 2 | GTGATGCTGACTGCCGTG | GAGATGCCAGCTTCCAAGG | 181 bp |
| Exon 3 | GGGTCTGAAGCCACTCAAGG | GTGACCTCCACAGGGCAG | 217 bp |
| Exon 4 | AGCAGGGTGTATGCTCTTCC | CTCAGGAGGGGTGCTCG | 214 bp |
| Exon 5 | CTCCCTCAGCCCCACAC | GTCCTGTCCCCAACCTGG | 175 bp |
| Exon 6 | GTTCCTGGAGCCCGACAC | GTCCCCATGCCTGGAAG | 207 bp |
| Exon 7 | GTGGCTGACCACTGTCCC | AGCTGTCCAAGGAGCCAG | 184 bp |
| Exon 8 | GCAGTGGCCTGTGTGGAC | AAAATGACAGTGACCAGGGC | 177 bp |
| Exon 9 | GTGGCTCAGCAGGTGACAG | CTGCTAGCAAGAAGGCCC | 210 bp |
| Exon 10 | CAGGAACCGCTAATCTGTTG | AAAAGGCAGTGACCTTCCC | 232 bp |
| Exon 11 | GAGGCCTAACGTGCTGTCC | AGTCCCCTCCAGCCCAG | 200 bp |
| Exon 12 | ATGACCAGCACAGGGTGG | TGAGGGCAGGAAGGCTC | 159 bp |
| Exon 13 | GTCACTGCCTGCACTTTGAG | TGATCATGCAGCTGGGG | 239 bp |
| Exon 14 | AGGGCCAGGTGTGAACTG | GGGCCCAGAGTAACTGACAC | 117 bp |
| Exon 15 | CACTTGGCCCTGATTTGG | GGCCCCAGGACGCTAAC | 147 bp |
| Exon 16.1 | GCTTCCCACCACTGACTCTC | GTTGCTGGGCAGGAAGAG | 219 bp |
| Exon 16.2 | TCTCCTTGCACGGTGGC | CTCTCACTCAGGCCCATCC | 219 bp |

**Supplementary table 2a: Multiplex PCR 1**

|  | Volume of 25 μl |
| --- | --- |
| A. dest. | 14.25 μl |
| 10xAccuPrime PCR Buffer II (Invitrogen, Thermo Fisher Scientific, Waltham, USA) | 2.5 μl |
| Primer E03 F/R | 1 μl |
| Primer E05 F/R | 1 μl |
| Primer E07 F/R | 1 μl |
| Primer E10 F/R | 2 μl |
| Primer E12 F/R | 1 μl |
| AccuPrime Taq DNA Polymerase High Fidelity (Invitrogen, Thermo Fisher Scientific, Waltham, USA) | 0.25 μl |
| DNA (20ng/μl) | 2 μl |

All primers have a concentration of 10 μM

**Supplementary table 2b: Multiplex PCR 2**

|  | Volume of 25 μl |
| --- | --- |
| A. dest. | 13.75 μl |
| 10xAccuPrime PCR Buffer II (Invitrogen, Thermo Fisher Scientific, Waltham, USA) | 2.5 μl |
| Primer E02 F/R | 1 μl |
| Primer E04 F/R | 1 μl |
| Primer E09 F/R | 1 μl |
| Primer E11 F/R | 0.5 μl |
| Primer E14 F/R | 2 μl |
| Primer E16.1F/16.2R | 1 μl |
| AccuPrime Taq DNA Polymerase High Fidelity (Invitrogen, Thermo Fisher Scientific, Waltham, USA) | 0.25 μl |
| DNA (20ng/μl) | 2 μl |

All primers have a concentration of 10 μM

**Supplementary table 2c: Multiplex PCR 3**

|  | Volume of 25 μl |
| --- | --- |
| A. dest. | 11.92 μl |
| 10xAccuPrime PCR Buffer II (Invitrogen, Thermo Fisher Scientific, Waltham, USA) | 2.5 μl |
| Primer altE01F/R | 0.33 μl |
| Primer E06 F/R | 2 μl |
| Primer E08 F/R | 1 μl |
| Primer E13F/R | 2 μl |
| Primer E15 F/R | 2 μl |
| Primer E16.1 F/R | 1 μl |
| AccuPrime Taq DNA Polymerase High Fidelity (Invitrogen, Thermo Fisher Scientific, Waltham, USA) | 0.25 μl |
| DNA (20ng/μl) | 2 μl |

All primers have a concentration of 10 μM

**Supplementary table 2d: Conditions of multiplex PCR**

| 94°C | 02:00 min |
| --- | --- |
| 35 cycles: 94°C | 00:15 min |
| 60°C | 00:15 min |
| 68°C | 01:00 min |
| 72°C | 20:00 min |
| 15°C | forever |

**Supplementary table 3a: PCR exon 1**

|  | Volume of 25 μl |
| --- | --- |
| A. dest. | 13.5 μl |
| GC-Rich Buffer A (Invitrogen, Thermo Fisher Scientific, Waltham, USA) | 5 μl |
| Primer E01 full F | 2 μl |
| Primer E01 full R | 2 μl |
| AccuPrime GC-Rich DNA Polymerase (Invitrogen, Thermo Fisher Scientific, Waltham, USA) | 0.5 μl |
| DNA (20ng/μl) | 2 μl |

All primers have a concentration of 10 μM

**Supplementary table 3b: Nested PCR exon 1**

|  | Volume of 25 μl |
| --- | --- |
| A. dest. | 14.5 μl |
| GC-Rich Buffer A (Invitrogen, Thermo Fisher Scientific, Waltham, USA) | 5 μl |
| Primer F (E1.1/1.2/1.3) | 2 μl |
| Primer R (E1.1/1.2/1.3) | 2 μl |
| AccuPrime GC-Rich DNA Polymerase (Invitrogen, Thermo Fisher Scientific, Waltham, USA) | 0.5 μl |
| PCR product of exon 1 full | 1 μl (dilution: 1:10) |

All primers have a concentration of 10 μM

**Supplementary table 3c: Conditions of PCR E01 and nested PCR**

| 95°C | 03:00 min |
| --- | --- |
| 35 cycles: 95°C | 00:30 min |
| 59°C | 00:30 min |
| 72°C | 01:30 min |
| 72°C | 10:00 min |
| 15°C | forever |

**Supplementary table 4a: A-tailing of the PCR products**

|  | Volume of 60 μl |
| --- | --- |
| NEBNext Ultra II End Prep Enzyme Mix (New England Biolabs, Ipswich, USA) | 3 μl |
| NEBNext Ultra II End Prep Reaction Buffer (New England Biolabs) | 7 μl |
| Pooled PCR product | 50 μl |

**Supplementary table 4b: Conditions of A-tailing**

| 20°C | 30:00 min |
| --- | --- |
| 65°C | 30:00 min |
| 4°C | forever |

**Supplementary table 5a: Annealing and phosphorylation of the oligo adapters**

|  | Volume of 10 μl |
| --- | --- |
| Oligo adapter 1 | 1 μl |
| Oligo adapter 2 | 1 μl |
| 10x Puffer A (New England Biolabs, Ipswich, USA) | 1 μl |
| ATP | 1 μl |
| T4 Polynucleotide Kinase (New England Biolabs) | 0.5 μl |
| ddH_2_O | 5.5 μl |

**Supplementary table 5b: Conditions of annealing and phosphorylation of the oligo adapters**

| 37°C | 30:00 min |
| --- | --- |
| 95°C | 05:00 min |
| Ramp down to 25°C at 5°C/min |  |

**Supplementary table 6: Ligation of oligos and PCR products**

|  | Volume of 20 μl |
| --- | --- |
| Oligo adapters (annealed and phosphorylated) (dilution 1:20) | 3.3 μl |
| Pooled PCR product | 100 ng |
| ddH_2_O | Fill up to 10 μl |
| Blunt/TA Ligase MasterMix (New England Biolabs, Ipswich, USA) | 10 μl |

Incubate for 45 minutes at room temperature

**Supplementary table 7a: Index PCR**

|  | Volume of 50 μl |
| --- | --- |
| Pooled PCR product | 20 μl |
| Nextera Index 1 Primer (N7xx) (Illumina, San Diego, USA) | 5 μl |
| Nextera Index 2 Primer (E5xx) (Illumina, San Diego, USA) | 5 μl |
| KAPA Hifi Ready Mix (Roche Sequencing and Life Science, Kapa Biosystems, Wilmington, USA) | 20 μl |

**Supplementary table 7b: Conditions of index PCR**

| 95°C | 03:00 min |
| --- | --- |
| 12 cycles: 98°C | 00:30 min |
| 60°C | 00:30 min |
| 72°C | 01:00 min |
| 72°C | 10:00 min |
| 15°C | forever |
